# An evaluation of the efficacy of a supplemental computer-based tutorial to enhance the informed consent process for cataract surgery: an exploratory randomized clinical study

**DOI:** 10.1186/s12886-022-02652-z

**Published:** 2022-11-11

**Authors:** Marlies Ullrich, Oliver Findl, Katharina Kefer, Birgit Döller, Ralph Varsits, Julius Hienert, Nino Hirnschall

**Affiliations:** Vienna Institute for Research in Ocular Surgery (VIROS), A Karl Landsteiner Institute, Hanusch Hospital, 1140 Vienna, Austria

**Keywords:** Informed consent, Cataract surgery, Audio-visual tutorial, Patient education

## Abstract

**Background:**

To assess whether informing patients with a computer-based tutorial in addition to standard informed consent influences the patient’s attitude towards surgery and increases patient’s knowledge.

**Methods:**

In this prospective, exploratory, randomized clinical study, patients scheduled for their first eye cataract surgery were randomly allocated to two groups, receiving standard face-to-face informed consent (control group) or additionally using an interactive computer-based tool (CatInfo) containing an audiovisual presentation about cataract and its treatment (study group). Cataract-related knowledge and decisional confidence (decisional conflict scale (DCS)) were assessed as well as one-month postoperatively decisional regret (decision regret scale (DRS)) and willingness to exchange face-to-face discussion time for the use of such a tool.

**Results:**

The study comprised 134 patients, 64 patients in the study group and 70 in the control group. Patients in the study group answered more questions correctly, 16.3 ± 2.0 (median 16.5, 11.0–19.0) versus 15.5 ± 1.9 (median 16.0, 8.0–19.0; *p* = 0.01). Patients showed a high decisional confidence with a study group mean DCS score of 92.4 ± 9.8 (median 96.9, 65.6–100) and control group score of 91.6 ± 10.9 (median 95.3, 43.3–100; *p* = 0.52). Mean DRS score in the study group was 2.5 ± 8.0 (median 0, 0–40) and 4.3 ± 12.5 (median 0, 0–75) in the control group (*p* = 0.14). Of study group patients 23 (67.6%) were willing to trade time, on average 158 ± 180 s (median 120 s, 45–900). Satisfaction with the tool was high with a mean of 9.1 ± 1.3 out of 10 (median 9.7, 5.0–10).

**Conclusions:**

Cataract-related knowledge was generally good, with slightly higher scores in the study group. In both groups, decisional confidence was high and regret after surgery was low. A tendency towards slightly higher decisional confidence and lower regret was found in the study group, although these differences were not statistically significant. Additional use of an interactive computer-based tool may prove useful in the informed consent process in a high-volume cataract outpatient setting.

**Trial registration:**

ClinicalTrials.gov, NCT04975126. Retrospectively registered – July 23, 2021.

**Supplementary Information:**

The online version contains supplementary material available at 10.1186/s12886-022-02652-z.

## Introduction

Before every non-emergency surgical procedure, as for cataract surgery, informed consent needs to be obtained within a personalized face-to-face discussion between the patient and qualified medical staff. This needs to cover the diagnosis, the procedure, risks, complications, benefits and alternatives as well as the consequences of refusing the recommended procedure to enable a patient to make an educated decision [1]. The requirements concerning the informed consent process are high. The amount of information needed to be explained to the patient is growing, as procedures get more complex and the number of treatment options increase. In the routine clinical setting, particularly in public health care, time pressure can be a limiting factor. Despite these challenging circumstances, at the end of the informed consent process patients should feel well informed and supported, ready to make an informed decision. Apart from the importance of a successful informed consent process for the physician–patient relationship, trust and patient satisfaction, the informed consent procedure and its adequate documentation can also play an important role when it comes to malpractice litigations. Having a satisfied and educated patient through an effective informed consent reduces the risk of malpractice litigation [2]. The largest share of malpractice claims out of all ophthalmic subspecialties seems to result from cataract surgery [3–5]. When it comes to litigation, surgical mistakes are often difficult to prove and therefore in most cases insufficient informed consent taking is implicated as an additional cause of dispute [6, 7].

Information from a face-to-face discussion may be poorly retained and also printed information material may not adequately solve this problem [8, 9]. For instance, difficult expressions and explanations or poor functional literacy of patients can be an obstacle.

A multi-media assisted informed consent process seems to be on the rise and several studies have already shown its benefit in informed consent taking for cataract surgery [10–15]. Most studies showed that cataract-related knowledge was significantly better when a multi-media device was additionally used [10–13]. The time for the consent process could be reduced [14, 15]. Zhang et al. showed higher patient satisfaction in the multi-media assisted group [14], others did not show a statistically significant difference [11, 15]. There was the concern that use of video material and a more detailed knowledge of the procedure could lead to increased anxiety [16]. Instead of an increase, either lower anxiety levels in the video-assisted group or no significant difference between the groups were found [11, 12]. For the computer-based tutorial CatInfo, the knowledge of patients was significantly higher, as demonstrated in a randomized, triple masked clinical trial [10]. The CatInfo tool is an audiovisual presentation on a tablet with headphones, which was developed by ophthalmologists of this research group and graphic designers. It covers the topics cataract, cataract surgery, risks, and complications.

The present study aimed to focus on the influence of the CatInfo tool on the decisional conflict to undergo cataract surgery and decisional regret. Furthermore, the general preference of involvement in treatment decisions, cataract-related knowledge, satisfaction with the tool and willingness to trade face-to-face informed consent discussion time for the use of such a tool in a future informed consent process were assessed.

## Methods

This exploratory, prospective, randomized study was performed at the Vienna Institute for Research in Ocular Surgery (VIROS) at the Department of Ophthalmology of the Hanusch Hospital, Vienna, between April 2015 and March 2017. For this study, patients aged above 18 years with age-related cataract scheduled for cataract surgery of their first eye to be performed under local anesthesia were recruited. Patients who had undergone previous ophthalmic surgery and/or had a visual acuity (VA) of less than 0.1 Snellen in the worse eye were excluded. Further exclusion criteria included dementia, depression, anxiety disorders, severe hearing loss and inability to use a touch screen device. To rule out undiagnosed memory disorders, all participants underwent the abbreviated mental test score (AMTS) [17]. The maximum score that can be reached is 10. Patients with an AMTS of less than 7 were not enrolled. Patients who were medically qualified and potentially had prior knowledge about cataract surgery were also excluded.

The study was conducted in accordance with the principles of the Helsinki Declaration and approved by the ethics committee of the city of Vienna (EK 14–250-0115). Written informed consent was obtained prior to enrolment in the study. The study was retrospectively registered at ClinicalTrials.gov with the number NCT04975126 (23/07/2021).

Patients were recruited and consented at the beginning of the pre-assessment visit one week prior to surgery. Randomization (1:1) was performed using an online randomization tool (list randomization) [18]. Patients were allocated to the 2 groups by the VIROS study personnel according to the randomization list. Patients in the intervention group used the CatInfo tool prior to a face-to-face discussion with a clinician whereas the participants of the control group underwent the standard informed consent procedure only. In the CatInfo group, the face-to-face discussion was guided by the CatInfo feedback printout (explained below). In our hospital an informative booklet as well as the informed consent form are sent to patients via mail before their pre-assessment visit. After the face-to-face informed consent discussion and if there were no further questions, the patient and the physician signed the informed consent form.

Before the informed consent procedure, patients completed the Degner control preferences scale (CPS) assessing the patient’s preference for involvement in health care decision-making [19]. Five different roles ranging from the patient making the treatment decision to the sharing of responsibility to leaving the physician all decisions are described on cards consisting of a statement and a cartoon. Those 5 cards (A-E) needed to be put in preferred order according to the patient’s agreement. There are different options for scoring. Out of the 120 possible combinations, only 11 are transitive permutations, resulting in a score of 1 to 11 [20]. One means the most active role in the decision and 11 the most passive role. Also, the most preferred option was documented and analyzed.

After the informed consent procedure, all patients were asked to fill in a multiple-choice question (MCQ) questionnaire to assess the patient’s knowledge about cataract, cataract surgery, its risks and complications and postoperative care. Details on how the questionnaire was developed and validated has been described previously [10]. The questionnaire consisted of 23 multiple-choice questions, 15 with 4 possible answers (1 point each) and 8 with 2 possible answers (0.5 points each). Therefore, the highest possible score was 19. The questionnaire also included questions about the highest level of education reached and about computer usage. For study group patients it also comprised visual analogue scales (VAS) to evaluate the CatInfo tool (0—not satisfied to 10—very satisfied) and whether the patient would like to use a similar tool before another surgery (0—no, certainly not to 10—yes, sure).

To assess the decisional confidence regarding the treatment decision to undergo cataract surgery after the informed consent procedure, the decisional conflict scale (DCS) questionnaire was used [21]. The DCS is a tool to assess and quantify the patient’s uncertainty, factors contributing to uncertainty as well as the quality of the decision made [22, 23]. The German version translated and validated by Buchholz et al. was used for this study [24]. The score can be calculated in two different ways [24]. In this study, it was calculated as decisional confidence, also called decisional comfort, where 0 represents the lowest decisional confidence and 100 the highest decisional confidence. Additionally, three different subscales of the DCS were evaluated: certainty subscale, informed subscale and support subscale. Zero means the person feels extremely uncertain, uninformed, unsupported in decision-making and 100 the person feels extremely certain, informed and supported. In the DCS score analysis, if one question was not answered or more than one option was selected, this question was omitted and the score was calculated based on 15 items. If more than one item was missing a score was not calculated.

One month after surgery the patients’ satisfaction with their decision was evaluated with a German version of the decision regret scale (DRS) questionnaire via telephone interview. The DRS measures “remorse or distress over a (health care) decision”, in this case the decision to undergo cataract surgery, and consists of 5 items [25]. A DRS score of 0 means no regret, a score of 100 high regret [26]. The analysis was performed in three different ways: including (a) all patients, (b) only those without any kind of preexisting ocular comorbidity at the pre-assessment visit and (c) those without any preexisting comorbidities, potentially affecting VA after surgery. To evaluate the value of the CatInfo tool, the willingness to trade face-to-face informed consent discussion time for the use of such a tool in the future was assessed in study group patients. Patients of the study group were asked if in a future informed consent process for another surgery, they would be willing to exchange face-to-face consultation time with the physician in order to use a tool such as the CatInfo tool. If they responded with yes, they were asked how much time they would be willing to trade (trade-off time). If the patients did not understand despite repeated explanations or did not want to answer the question, this was counted as a missing response.

### The CatInfo tool

The CatInfo tool is a computer-based tutorial about cataract and cataract surgery run on a handheld device with headphones and presented in an audio-visual fashion. It covers the following topics: cataract, the surgical procedure of cataract surgery, risks and complications. The tutorial is split into 7 small chapters. To ensure the patient has understood every chapter a “traffic light” system is used: green – content understood, ready to continue; yellow – questions that require discussion with the physician and red – repetition of the module required. A printout with the results of the patient’s responses to the traffic lights is given to the physician giving informed consent. The tool has been described in detail in a previous publication by this group [10]. Screenshots of the CatInfo tool can be found in Additional file 1.

### Statistical analysis

For the statistical analysis SPSS software version 26.0 (IBM Corp., USA) was used. Descriptive data values are presented as mean, standard deviation (SD), median and range. Using the Kolmogorov–Smirnov test all data samples were first tested for normal distribution. In case of normally distributed data an independent sample t-test was used to compare the groups, in case of not normally distributed data a Mann–Whitney U Test was used. For nominal and ordinal parameters, a Chi-square test was performed. For all statistical tests the significance level was *p* < 0.05.

## Results

The study comprised 134 patients, 64 patients in the study group and 70 in the control group. Of the 150 patients included initially, 16 withdrew consent or had to be excluded (major protocol deviation including rescheduled surgeries (*n* = 7), withdrawal of consent (*n* = 2), exclusion due to compliance issues with the questionnaire completion (*n* = 7)). The distribution of age, sex, AMTS Score, highest educational level, computer usage and preexisting ocular comorbidities are outlined in Table 1. There were no statistically significant differences between the groups. Overall, 93 (69.4%) of patients had a computer at home. Forty-seven (35.1%) and 23 (17.2%) stated that they used a computer several times a day or a week respectively, 6 (4.5%) several times a month, 19 (14.2%) rarely and 39 (29.1%) had never used a computer before.

**Table 1:** Patient characteristics by group

	Group		*p* value*
	Study	Control	
n	64	70	
Age (years)			0.41§
Mean ± SD	69.5 ± 8.8	70.7 ± 8.2	
Median (range)	70.5 (51–91)	72.0 (48–93)	
Female (%)	53.1%	54.3%	0.89‡
AMTS	9.72	9.79	0.50§
Education level^¶^			0.33‡
No school leaving qualification	1 (1.6%)	2 (2.9%)	
Secondary school	12 (19.0%)	18 (25.7%)	
Apprenticeship	16 (25.4%)	14 (20.0%)	
Vocational school	20 (31.7%)	13 (18.6%)	
High school	7 (11.1%)	15 (21.4%)	
University degree	7 (11.1%)	8 (11.4%)	
Computer usage			0.53‡
Never used before	16 (25.0%)	23 (32.9%)	
Rarely	9 (14.1%)	10 (14.3%)	
Several times a month	3 (4.7%)	3 (4.3%)	
Several times a week	9 (14.1%)	14 (20.0%)	
Several times a day	27 (42.2%)	20 (28.6%)	
Ocular comorbidities	20 (31.3%)	19 (27.1%)	0.60‡
Glaucoma	7 (10.9%)	6 (8.6%)	
Amblyopia^a^	1 (1.6%)	2 (2.9%)	
Epiretinal membrane/vitreomacular traction^a^	1 (1.6%)	2 (2.9%)	
Age-related macular degeneration^a^ (nonexudative/exudative)	5 (7.8%) (5/0)	3 (4.3%) (2/1)	
Pseudovitelliform macular lesion^a^	0 (0.0%)	1 (1.4%)	
Corneal scar/opacification^a^	1 (1.6%)	3 (4.3%)	
Cornea guttata^a^	2 (3.1%)	1 (1.4%)	
Strabismus	0 (0.0%)	1 (1.4%)	
Synchysis	1 (1.6%)	1 (1.4%)	
Treated retinal tear	3 (4.7%)	1 (1.4%)	
Optic disc drusen	0 (0.0%)	1 (1.4%)	
Unilateral optic atrophy due to optic nerve sheath meningioma^a^	1 (1.6%)	0 (0.0%)	
Potential VA-related comorbidities	11 (17.2%)	12 (17.1%)	1.00‡

^* ^Comparison between the groups

^§^ Mann–Whitney U Test

^‡^ Pearson Chi Square Test

^¶ ^One missing value in study group

^a ^Considered as potential VA-related comorbidities

**Fig. 1:**
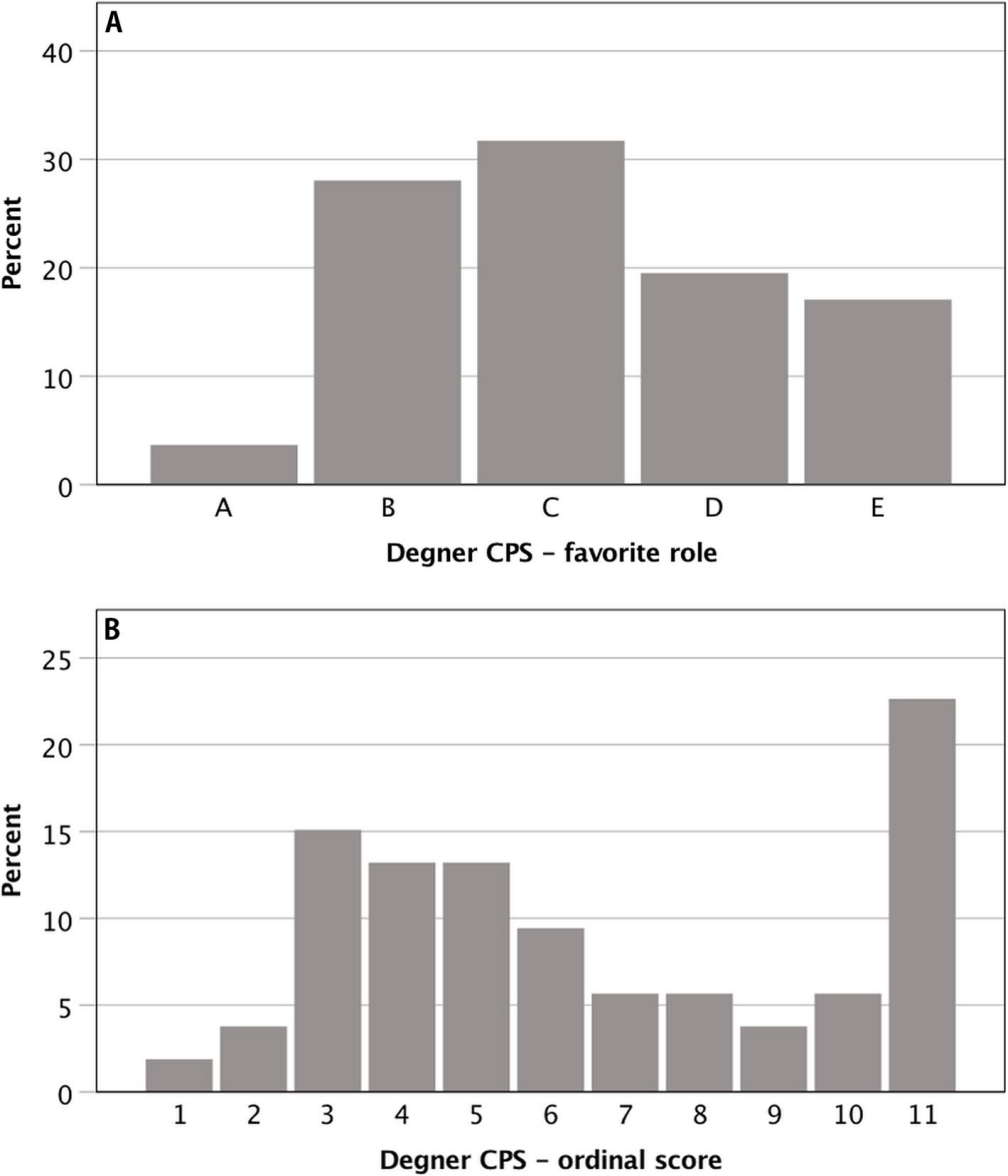
Degner control preferences scale: Panel **A** – favorite role of the patient: A—most active patient role, C—collaborative decision, E—most passive patient role, *n* = 82; Panel **B** – ordinal scale: 1—the most active role in the decision; 11—most passive role; only transitive permutations counted, *n* = 53 (CPS = control preferences scale)

The most preferred role in the Degner CPS was sharing of responsibility (role C) by 26 patients (31.7%). Fifty-three (64.6%) patients put the cards of the Degner CPS in a logical order according to their preferred involvement in decision-making (corresponding to one of the 11 valid transitive permutations, see Fig. 1).

**Fig. 2:**
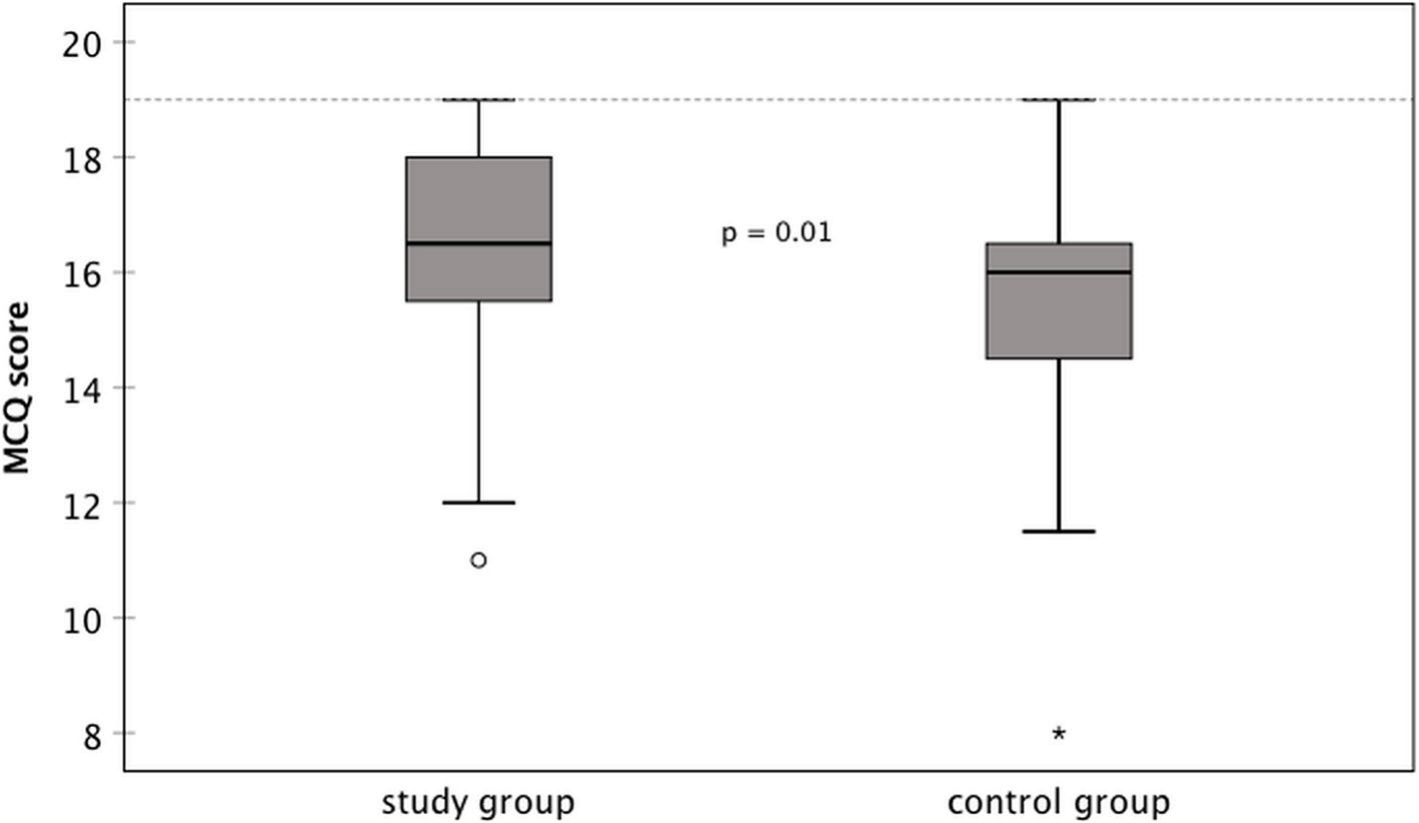
Knowledge on cataract and cataract surgery at the pre-assessment visit measured by correctly answered MCQs; maximum score 19 points (dashed line), *n* = 134 (MCQ = multiple-choice question)

As shown in Fig. 2 both groups scored well on the MCQ assessing cataract-related knowledge, with only a few outliers. The study group and the control group achieved a mean MCQ score of 16.3 ± 2.0 (median 16.5, 11.0 to 19.0) and 15.5 ± 1.9 (median 16.0, 8.0 to 19.0), respectively. This difference was statistically significant (*p* = 0.01).

Patient satisfaction with the CatInfo tool was high with a mean score of 9.1 ± 1.3 out of 10 (median 9.7, 5.0 to 10; *n* = 62). A mean score of 9.3 ± 1.1 out of 10 (median 9.9, 5.2 to 10; *n* = 62) was reached on the VAS whether the patient would like to use a similar tool before another surgery.

**Fig. 3:**
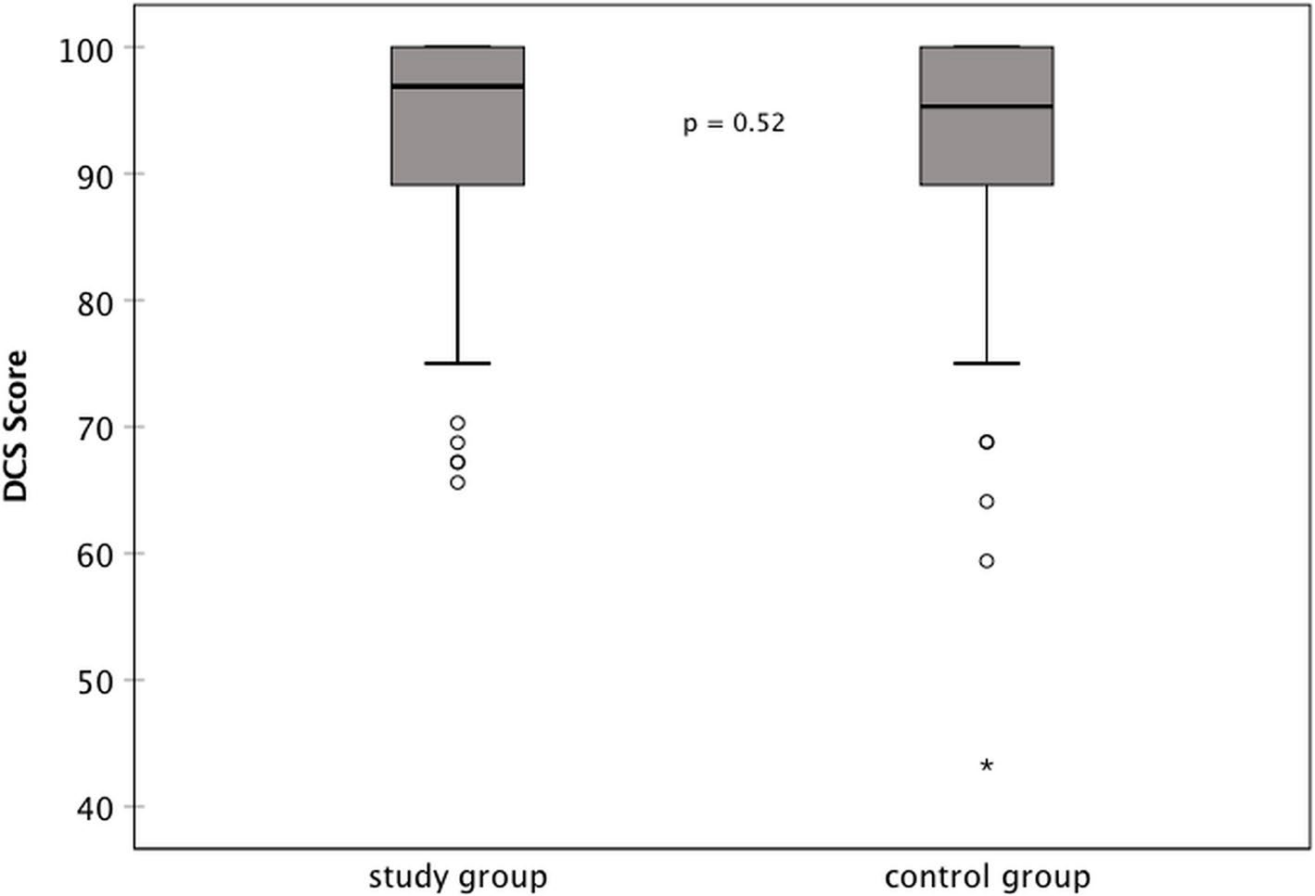
Decisional conflict scale; results shown here as decisional confidence, 0—lowest decisional confidence, 100—highest decisional confidence, *n* = 131 (DCS = Decisional conflict scale)

Overall patients showed a high decisional confidence of 92.0 ± 10.4 out of 100 (median 95.3, 43.3 to 100). The decisional confidence was similar between the groups (*p* = 0.52) with a mean DCS score of 92.4 ± 9.8 (median 96.9, 65.6 to 100) in the study group and 91.6 ± 10.9 (median 95.3, 43.3 to 100) in the control group (Fig. 3). Results of the certainty subscale, informed subscale and support subscale are shown in Table 2.

**Table 2:** Results of the DCS subscales of interest

	Group		*p* value*
	Study	Control	
n	62	69	
Certainty subscale			
Mean ± SD	91.5 ± 13.4	91.3 ± 14.2	0.98
Median (range)	100 (50.0-100)	100 (41.7-100)	
Informed subscale			
Mean ± SD	90.3 ± 17.0	87.9 ± 19.0	0.42
Median (range)	100 (16.7-100)	100 (8.3-100)	
Support subscale			
Mean ± SD	91.1 ± 13.7	89.9 ± 16.8	0.94
Median (range)	100 (50.0-100)	100 (25.0-100)	

*DCS* Decisional conflict scale

0—feels extremely uncertain, uninformed, unsupported

100—feels extremely certain, informed, supported

^* ^Comparison between the groups, *p* values calculated with Mann–Whitney U Test

Intraoperatively, there was one case of zonulolysis, which could be handled with a capsular tension ring (study group), one case with an IOL exchange due to a damaged IOL haptic (study group), one case of a capsular defect without vitreous prolapse, which was transformed into a posterior continuous curvilinear capsulorhexis with IOL implantation in the bag (control group), and one case of radial anterior capsular tear (control group). Those patients had a normal postoperative course without any sequelae.

With regard to the decisional regret analysis, only cases without serious complications in the intra- and postoperative period could be included. In one case, a patient had a retinal detachment surgery (vitrectomy, endolaser and silicone oil tamponade) 15 days after cataract surgery. This patient was excluded from this part of the analysis. Overall, 94 (82.5%) patients scored 0 out of 100 on the DRS (i.e. no regret) and 103 (90.4%) scored ≤ 10, one month after surgery (*n* = 114). In the study group 47 of 53 (88.7%) scored 0 on the DRS, in the control group 47 of 61 (77.0%). The mean DRS score in the study group was 2.5 ± 8.0 (median 0, 0 to 40) and 4.3 ± 12.5 (median 0, 0 to 75) in the control group. This was not statistically significant (*p* = 0.14). After excluding preexisting ocular comorbidities and also after only excluding potential VA-related comorbidities, there was no statistically significant difference in the DRS score between the groups: study group 2.3 ± 7.9 (median 0, 0 to 40) versus control group 3.4 ± 12.0 (median 0, 0 to 75; *n* = 85; *p* = 0.35) and 2.0 ± 7.5 (median 0, 0 to 40) versus 3.5 ± 11.4 (median 0, 0 to 75; *n* = 97; *p* = 0.18) respectively.

Patients without any ocular comorbidities scored 2.9 ± 10.2 (median 0, 0 to 75; *n* = 85) and with ocular comorbidities 5.2 ± 11.8 (median 0, 0 to 55); *n* = 29), without comorbidities, potentially affecting VA 2.8 ± 9.8 (median 0, 0 to 75; *n* = 97) and with 7.4 ± 14.5 (median 0, 0 to 55; *n* = 17), respectively.

**Fig. 4:**
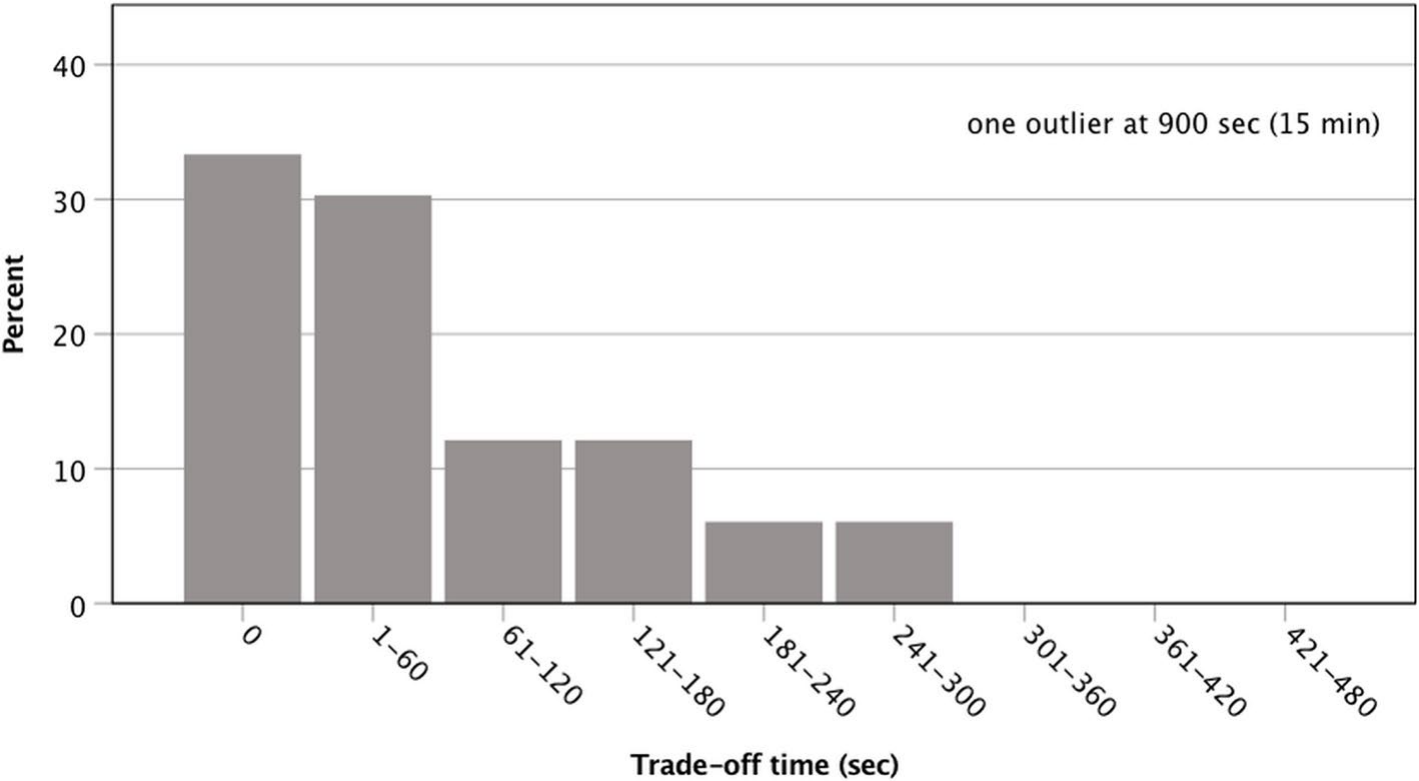
Trade-off times in categories of one minute; 32.4% were not willing to trade time (trade-off time 0 s), 67.6% were willing to trade time, one patient was willing to trade 15 min (not shown in bar chart), *n* = 34

In total, 23 (67.6%) of study group patients answered that they would be willing to trade time of their face-to-face informed consent discussion for using such a tool in a future informed consent process. In those patients, who were willing to trade time, the traded time was on average 158 ± 180 s (median 120 s, 45 to 900), see Fig. 4.

## Discussion

The present study was conducted to assess the effect of an interactive computer-based multimedia tool on the patient’s attitude towards surgery, in particular, on the decisional conflict to undergo cataract surgery and decisional regret as well as on cataract-related knowledge.

As part of assessing the patient’s attitude towards the surgery, the patient’s wish to generally become involved in health-care decisions was evaluated by Degner CPS. The Degner CPS was primarily designed and used for patients with serious illnesses, but is widely used for assessing the preference for involvement in health care decision-making [19, 27]. At the beginning of the study, organizational issues occurred in the conduction of the Degner CPS. Therefore, an amendment was submitted to the ethics committee to increase sample size. The Degner CPS was only performed in 82 patients. The most preferred role was the collaborative role (31.7%). Combining the two active and two passive roles leads to a rather balanced result with a tendency towards the more passive roles, active 31.7%, collaborative 31.7% and passive 36.6%. In 64.6% the preferred order of roles corresponded to one of the 11 transitive permutations. In this study, for the ordinal score only those 11 transitive permutations were counted. Of those with a valid score, 22.6% scored 11, corresponding to the most passive patient role. A broader secondary peak can be seen at a score of 3–5, together representing 41.5% and corresponding to an active-collaborative role.

In this study, patients who used a computer-based tutorial additional to the standard informed consent procedure showed a slightly higher cataract-related knowledge in the MCQ questionnaire. This confirms the finding of a previous study using the CatInfo tool [10]. However, there was a higher difference between the groups in the previous study due to the difference in study design. In this study, the physician giving the informed consent received the printout of the CatInfo tool to guide him/her through the face-to-face discussion. In contrast, in the previous study the physician was masked and did not know, if the patient had received additional information through the use of the CatInfo tool [10].

Overall patients of both groups performed quite well on the MCQ. The mean score in both groups was higher than in the previous study, showing a mean score of 15 in the study group and 12 in the control group [10]. The study population in the previous study was slightly older than the one of this study (study group 73 versus 70 years; control group 75 versus 71 years). Another reason could be that, due to the fact that the other study was conducted several years earlier, the internet is now even more widely used among all age groups and, thereby, patients may have easier access to information material. Patients, especially younger ones, sometimes seem surprisingly well informed already on arrival for their pre-assessment visit, for example asking detailed questions on IOL types. However, one would expect that especially in the elderly, the use of the internet is not that widespread. In this study, only information on computer usage and not internet usage was collected. In our study population more than two thirds (69.4%) had a computer at home and more than half (52.3%) stated that they used a computer several times a day or a week; 4.5% several times a month, 14.2% rarely and 29.1% had never used a computer before. So, in the latter group use of Internet seems very unlikely.

A study assessing the quality content of educational cataract videos on “YouTube” has shown that the majority of videos were not adequately educational, also containing biased information with a commercial background [28]. Information material on websites has also been assessed regarding the readability. Studies have shown that online patient education material in ophthalmology was written above the recommended reading level for the average population, also by renowned ophthalmological societies [29–31]. So, there is concern that online contents are adequately presented and correctly understood by patients. The advantage of tools and videos that are shown before the face-to-face discussions is that questions can be answered and misunderstandings may be resolved straight away. With the CatInfo tool the patient gives feedback after each chapter using a traffic light system described above to ensure that the content has been understood. A printout that summarizes what the patient has selected after each chapter immediately tells the physician which topics have been poorly understood or were unclear.

As mentioned above, we need to bear in mind that, due to the higher age in the majority of cataract patients, the internet may not have gained such an importance compared to other generations. In our sample nearly one third of patients have never used a computer before.

Another possible explanation for the high knowledge in both groups in our study could be that at our department patients receive quite detailed information material via mail well ahead to their pre-assessment visit. Additional to the informed consent form they receive a detailed booklet on cataract surgery. In this study, knowledge was only assessed after the face-to-face discussion. To also assess the pre-existing knowledge, it would have been necessary to fill in a questionnaire before the start of the informed consent procedure and thereafter.

The satisfaction with the tool was high. Interestingly, VAS scores were even higher regarding the question, whether they would want to use such a tool before another surgery. This could mean that the concept of this multi-media approach seems to be very well accepted and patients would appreciate an implementation of such tools beyond ophthalmology.

Our focus was to assess the patient’s attitude before and after surgery, and to explore whether multi-media assisted informed consent influences decisional conflict and decisional regret. Overall patients were shown to have a very low decisional conflict regarding their decision to undergo cataract surgery. We reported the score as decisional confidence, as explained by Buchholz et al. [24], with 100 meaning the highest decisional confidence and thereby the lowest decisional conflict. The overall score and also in the assessed subscales mean scores were slightly higher in the study group, but no statistically significant difference was found. The high decisional confidence in both groups could result from the physician being very informative and supportive when giving informed consent. The value of the CatInfo tool could be much higher when this is not the case.

The DCS has already been used for the assessment of the decisional conflict related to cataract surgery. Newman-Casey et al. assessed how non-physician pre-surgical counselors teaching patients in India influenced knowledge and the decisional conflict [32]. They did not use the current 16-items questionnaire for research purposes but used the original 9 questions and added 3 of the 7 new items. The score was also calculated differently. Comparing before and after the teaching, the decisional conflict score was shown to improve by 14%, meaning the decisional conflict decreased. In our study the decisional conflict was not assessed at the start of the pre-assessment visit, only after. Therefore, we cannot give any information on how much the informed consent process strengthened the decisional confidence.

The high decisional confidence scores in our study could have also resulted from a generally rather low decisional conflict, as patients are normally aware of the planned surgery several months in advance.

The DRS assessed the “remorse or distress” of the decision to undergo cataract surgery [25]. Overall, decisional regret was small. Mean scores were even lower in the study group, but not statistically significant. To assess the effect of the CatInfo tool on decisional regret, cataract surgery and the following postoperative period had to be without major complications. One patient suffered from a retinal detachment soon after the cataract surgery, before the 1-month telephone interview. Therefore, the score of that patient was excluded. Reasons for regret with the decision in patients with higher scores seem to have been dysphotopsia, glare and more intense perception of floaters after surgery. One patient with a DRS score of 55 had an epiretinal membrane already present at the pre-assessment visit, which was treated with pars plana vitrectomy and membrane peeling 6 months after cataract surgery. As postoperative VA and visual function influence the satisfaction with the decision and thereby regret, patients should ideally not have any additional ophthalmological conditions affecting vision or, if a more real-life study setting is preferred, randomization should be stratified for additional conditions. In this study, patients with other ocular diseases were included. All patients seen at the pre-assessment visit were referred for cataract surgery with a referral letter of their community ophthalmologist. Inclusion and randomization took place before the ophthalmological examination. Ocular comorbidities, that were recorded at the pre-assessment visit, were relatively evenly distributed without any statistically significant differences between the groups. Also, after exclusion of patients with comorbidities and exclusion of patients with comorbidities potentially affecting VA, no statistically significant difference was found in the DRS score between the study group and the control group. As expected, regret was higher in patients with comorbidities potentially affecting VA than in those without.

Measurement of VA as an outcome related to regret would have been an interesting measure, but the only postoperative follow-up visit in this study was performed via telephone interview. Another limitation is that patients were not systematically evaluated regarding issues after surgery such as dysphotopsia, that could influence regret after surgery. Only patients, who seemed to have greater regret, were asked about reasons for their regret and a note was made in the comment field.

More than two thirds of study group patients answered in the telephone interview that they would be willing to trade face-to-face informed consent discussion time in order to use such a tool in a future informed consent process. For some patients this question was rather difficult to understand, despite detailed explanations. This resulted in a relatively high number of missing responses, which poses a limitation.

## Conclusions

The additional use of an interactive computer-based multimedia tool can successfully improve the informed consent process. Cataract-related knowledge was generally good, with slightly higher MCQ scores in the study group. In the study group, a tendency towards slightly higher decisional confidence and lower regret was found, although these differences were not statistically significant. Overall decisional confidence was high and regret after surgery was low in both groups. A translated version of the tool in Turkish and Serbian has already been developed for patients not literate in German to further improve the informed consent process in those patient groups.

## Supplementary Information

**Additional file 1.**

## Data Availability

The datasets used and/or analyzed during the current study are available from the corresponding author on reasonable request.

## Abbreviations

AMTS: Abbreviated mental test score
CPS: Control preferences scale
DCS: Decisional conflict scale
DRS: Decision regret scale
MCQ: Multiple-choice question
SD: Standard deviation
VA: Visual acuity
VAS: Visual analogue scale
