# Supplementary figures and images for: An evaluation of the efficacy of a supplemental computer-based tutorial to enhance the informed consent process for cataract surgery: an exploratory randomized clinical study
Source: BMC Ophthalmol. 2022 Nov 11;22:430. doi: 10.1186/s12886-022-02652-z (PMC9650904; doi:10.1186/s12886-022-02652-z)

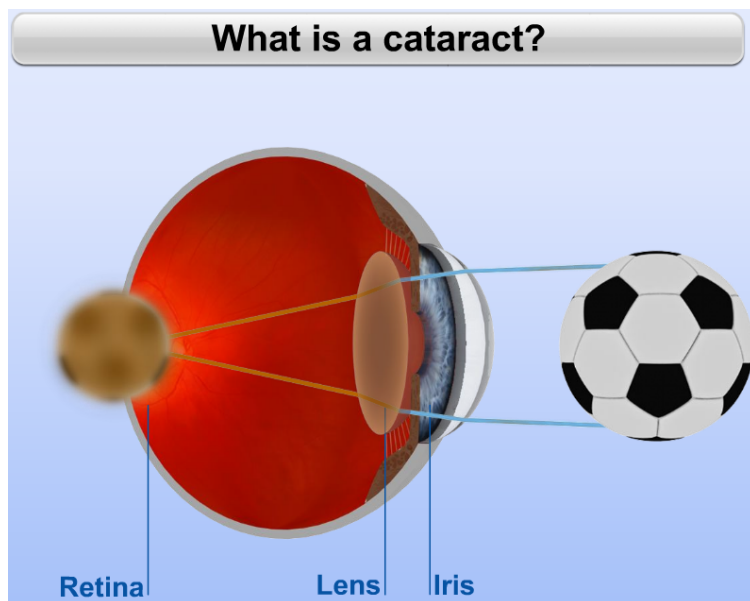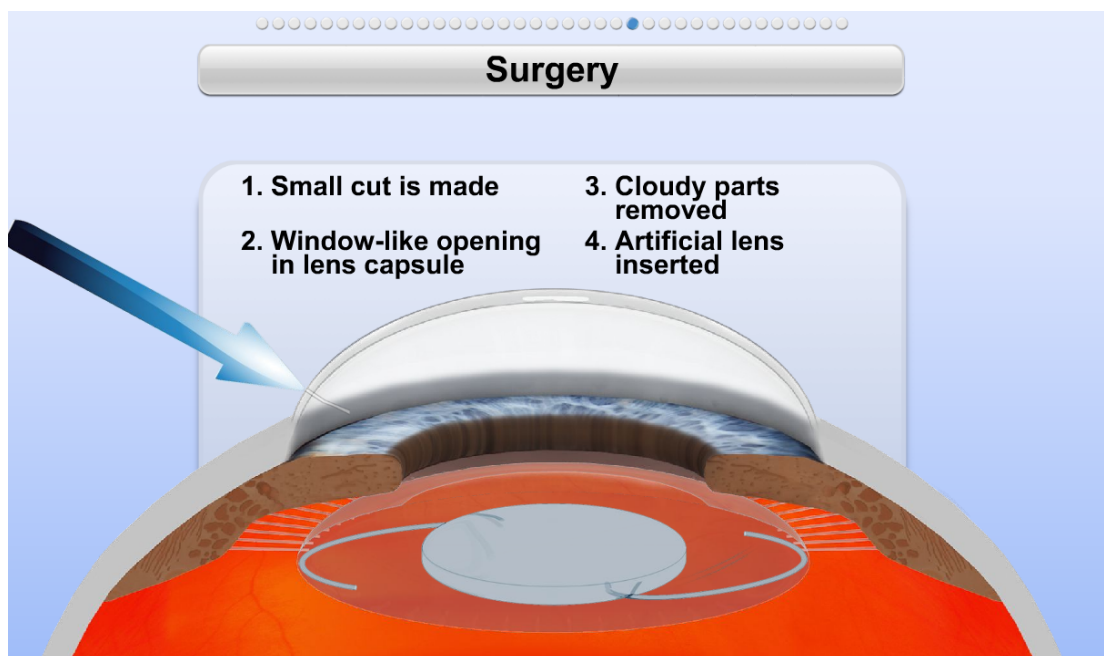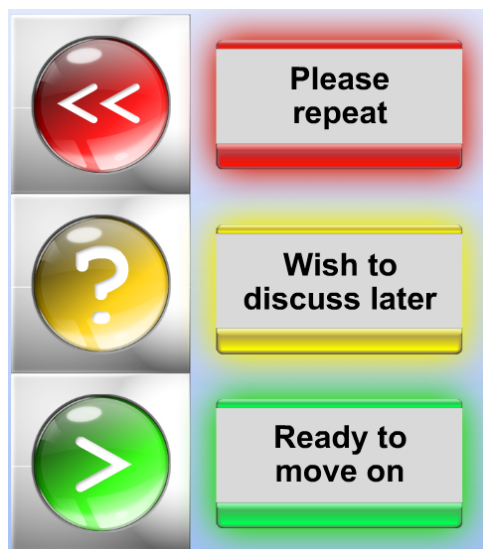

Supplement: Supplementary file 1 — Additional file 1. [file 12886_2022_2652_MOESM1_ESM.pdf]
